# *Toxoplasma gondii* in the Food Supply

**DOI:** 10.3390/pathogens6020021

**Published:** 2017-05-26

**Authors:** Malik A. Hussain, Victoria Stitt, Elizabeth A. Szabo, Bruce Nelan

**Affiliations:** Department of Primary Industries, NSW Food Authority, Newington, NSW 2127, Australia; Victoria.stitt@foodauthority.nsw.gov.au (V.S.); Lisa.Szabo@foodauthority.nsw.gov.au (E.A.S.); Bruce.Nelan@foodauthority.nsw.gov.au (B.N.)

**Keywords:** foodborne toxoplasmosis, cat feces, undercooked meat, unpasteurized milk, fresh plant products, contaminated water

## Abstract

Toxoplasmosis is caused by infection with the protozoan parasite *Toxoplasma gondii*. Infections are usually either asymptomatic or develop mild symptoms that are self-limited, but infections in immunosuppressed persons can be severe. Infections in pregnant women can cause serious health problems in the child such as mental retardation and blindness. Infection with *T. gondii* in immunocompetent adults can lead to impaired eyesight. Toxoplasmosis has ranked very highly in two studies of death and disability attributable to foodborne pathogens. The consumption of raw or undercooked meat containing *T. gondii* tissue cysts and the consumption of raw vegetables or water contaminated with *T. gondii* oocysts from cat feces is most frequently associated with human illness. The risk of acquiring a *Toxoplasma* infection via food varies with cultural and eating habits in different human populations.

## 1. Introduction

Toxoplasmosis is caused by infection with the protozoan parasite *Toxoplasma gondii*. There are three major genotypes (type I, type II, and type III) of this parasite that differ in their pathogenicity and prevalence in people [1]. For example, type II genotype is responsible for most cases of congenital toxoplasmosis in Europe and the USA [2]. Estimates suggest that 23% of adolescents and adults are infected with *T. gondii* [3], which accounts for 24% of deaths due to foodborne illness in the USA [4]. Although these infections are usually either asymptomatic or associated with self-limited symptoms (e.g., fever, malaise, and lymphadenopathy), infections in immunosuppressed persons (e.g., persons with acquired immunodeficiency syndrome [AIDS]) can be severe [5]. In addition, infections in pregnant women can cause serious health problems in the fetus if the parasites are transmitted (i.e., congenital toxoplasmosis) and cause severe sequelae in the infant (e.g., mental retardation, blindness, and neurological disorders) [6]. Infection with *T. gondii* is increasingly being recognized as a problem in non-pregnant, immunocompetent adults, where acute infection may lead to impaired eyesight [7].

*T. gondii* was ranked very highly in burden of disease estimates for foodborne pathogens in the Netherlands and the USA [8,9]. Congenital illness, resulting from infection of an unborn baby, can result in life-long disability. Infections in immune-compromised people can be severe. Eye problems can result from toxoplasmosis acquired later in life.

There are several routes by which *T. gondii* contamination of food and environment can directly or indirectly cause infection in human beings. High-risk food products include contaminated meat, unpasteurized goat’s milk, fresh plant products, and water [10]. Contaminated pets and wild cats, lawn grass, and garden soil could transmit this infection to humans. Recently, many publications have emerged that emphasize the role of food as a major transmission vehicle of this parasite: a document from Food Standards Australia New Zealand (FSANZ) on agents of foodborne illness was updated in 2014 [11]; Guo et al. [12] reviewed prevalence and risk factors for *T. gondii* infection in meat animals and meat products in 2015; and Hill and Dubey [8] discussed *T. gondii* as a parasite in food in 2016. This article provides a brief but comprehensive overview of the role of food supply in the infection of toxoplasmosis in humans.

## 2. Foodborne Toxoplasmosis

Toxoplasmosis can be transmitted to humans by three principal routes. First, humans can eat raw or inadequately cooked infected meat or eat uncooked foods that have come in contact with contaminated meat. Second, humans can inadvertently ingest oocysts that cats have passed in their feces, either in a cat litter box or outdoors in soil (e.g., soil from gardening or unwashed fruits or vegetables). Third, a woman can transmit the infection to her unborn fetus [13].

Water is increasingly being investigated as a risk factor [14] and has been demonstrated to be an important source of infection in tropical and subtropical countries, where surface water may be used for human consumption without any purification, but has also been found to be a risk factor in parts of Europe. The largest and best documented outbreak of acute toxoplasmosis in humans occurred in 110 individuals on Vancouver Island, Canada, in 1995. Comprehensive, retrospective epidemiological studies provided strong evidence that this outbreak was caused by the contamination of municipal drinking water with oocysts [7].

Toxoplasmosis has assumed interest since it ranked very highly in two studies of death and disability attributable to foodborne pathogens (Table 1). Havelaar et al. [8] found that *T. gondii* (congenital and acquired cases) caused the highest disease burden as measured by Disability Adjusted Life Years (DALY per year 2009). This fell to second place when an economic discount factor was applied, because the disability was very long term. The DALY per patient for congenital disease was second to perinatal *Listeria monocytogenes*. Approximately 45% of the total burden (for all the pathogens studied) was attributed to food. Hoffman et al. [9] ranked *T. gondii* second when measured by the estimated annual cost of illness and third in terms of losses of Quality Adjusted Life Years (QALY loss). These rankings used figures from a previous report that estimated toxoplasmosis cases to be 50% foodborne [15].

Despite the similarities in rankings, there are some large differences in the estimates for congenital and acquired/adult toxoplasmosis. While the papers agree on the significance of toxoplasmosis in total, they differ on the contribution of congenital and acquired. Deaths in congenital cases are estimated to be 13 in the Netherlands in 2009 [8] and 15 in the USA in 2009 [9]. Deaths in acquired *T. gondii* infections of adult cases were estimated to be 327 in the USA, whereas no case of death was reported in the Netherlands in 2009. Severe cases, requiring hospitalization, of adult toxoplasmosis were estimated to be 4428 in the USA.

Australian data for a range of foodborne pathogens was extracted by Kirk et al. [16]. They calculated there were 30 hospitalizations and one death due to toxoplasmosis annually between 2006 and 2010. These data are based on hospitalization data from state and territory health departments and deaths data from the Australian Bureau of Statistics. A multiplier of two was used to account for underreporting, just as it was in the USA estimates. The differences between USA, Dutch, and Australian hospitalization and death figures are hard to reconcile.

Petersen et al. [7] noted that the risk of acquiring a *T. gondii* infection via food varies with cultural and eating habits in different human populations. Even so, the differences between the three Western countries are pronounced. One possible problem with the USA estimates is the assumption that 15% of those that sero-convert will become symptomatic. Jones and Holland [17], reporting on ocular toxoplasmosis, estimated that 2% of infected people develop ocular lesions but only 0.2–0.7% develop symptomatic retinitis.

## 3. How Do Humans Become Infected with *T. gondii* through Food?

**Figure 1:**
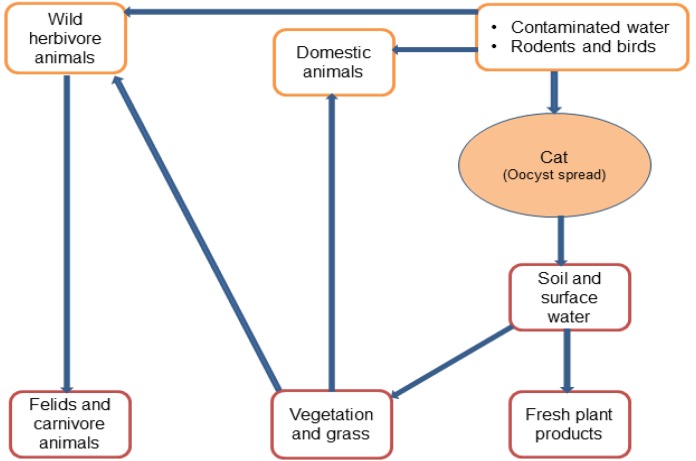
The spread of *T. gondii* in the environment and food supply chain.

Even though toxoplasmosis is the most prevalent parasitic zoonosis in humans, the system for routine monitoring and reporting is inadequate due to the difficulties in the detection of *T. gondii* infections via the foodborne route, as well as neglected and underreported cases [18]. There are still many unanswered questions about modes of transmission to humans and emerging risk factors, such as global sourcing of food and changing consumer vogues (for example, the increasing trend to consume raw vegetables and undercooked meat). Risk factor studies are needed in order to more accurately identify common sources of infections, including the environment. Figure 1 shows the spread of the parasite in different environments.

**Figure 2:**
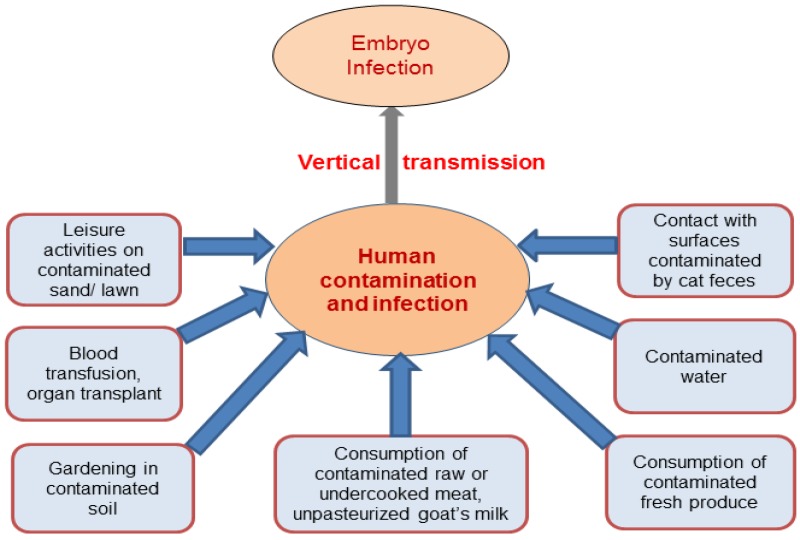
Direct sources of *T. gondii* infection in humans.

Several investigations have tried to determine which pathways of infection are important to humans, though the authors noted difficulties with these studies [7,19]. Figure 2 shows direct sources of *T. gondii* infection in humans. They tend to be small and focused on women of childbearing age, and risk factors for infection remain unexplained in a significant number of cases. The results are only applicable to the community where the studies were completed. Cook et al. reported a multi-center European study of 252 infected women and 708 controls [20]. They identified the following risk factors: raw sausage eaten at least once a week; dry cured meat eaten more than once a week; salami eaten more than once a week; raw/undercooked beef; raw/undercooked lamb; other raw/undercooked meats (venison, horse, rabbit, whale and game birds); tasting meat when cooking; unpasteurized milk; untreated water; contact with soil; working with animals; and travel outside of Europe/USA or Canada. In this and most other studies, contact with cats in the home was not identified as a risk of direct infection. Further evidentiary reports are listed in Table 2.

### 3.1. Raw and Undercooked Meat

A recent risk assessment suggests that meatborne *T. gondii* infection is a food safety concern. A considerable number of new *T. gondii* infections are associated with fresh pork consumption each year in the USA [21]. Raw/undercooked meat is identified as a significant risk but, as several authors note, this might change as animal friendly (organic, free-range) approaches to animal husbandry become more common. In Europe, between 30% and 63% of infections in the different centers could be attributed to meat consumption [20], but the type of meat differed (Table 2). Eating lamb and ‘other meat’ was more important in northern and central European centers than in Italy. The proportion of infections attributed to eating salami was 10–14% in Milan, Naples, and Brussels and 3–5% elsewhere. A qualitative risk assessment conducted by Guo et al. [22] concluded that exposure risks associated with meats from free-range chickens, non-confinement-raised pigs, goats, and lamb are higher than those from confinement-raised pigs, cattle, and caged chickens. Currently, it is difficult to draw a definite conclusion about the influence of the method of production on the prevalence of *T. gondii* in animal products, and further information and data is needed with conclusive evidence.

### 3.2. Milk and Milk Products

An American study by Jones et al. [30] looked at 148 *T. gondii* infected cases and 413 controls. This study confirmed the significance of several factors that had previously been linked with *T. gondii* infection, such as eating undercooked meat and drinking unpasteurized goat’s milk. They also recognized eating raw oysters, clams, or mussels as new risk factors. They also noted that exposure to three or more kittens increased the risk. This suggests that exposure to a litter of kittens may be responsible for the risk, especially when they have had access to rodents and birds. They were not able to explain the risk for 48% of the infections in their study. In Lausanne, 14% of infections were attributed to consumption of unpasteurized milk or milk products, whereas elsewhere it was 5% or less. In a European multicenter study, all research centers reported a large proportion of infections (from 14% to 49%) that remained unexplained by the exposures studied [19]. Although *T. gondii* have been found in the unpasteurized milk of sheep, goats, and cows, only drinking of unpasteurized goat’s milk poses an elevated risk of infection in human [27].

### 3.3. Fresh Plant Products and Water

*Toxoplasma* contamination of food can occur because of unwashed hands prior to food preparation after contact with plants or soil in the garden, a cat, cat feces, or the cat litter box. Commenting on 16 case-control studies, Petersen et al. noted that these studies failed to explain the high frequency of sero-positivity (24–47%) in some populations of vegetarians [7]. Water is recognized as a vehicle for disseminating *T. gondii* oocysts and investigations of waterborne toxoplasmosis are needed [14]. The ingestion of unfiltered water contaminated with *T. gondii* oocysts was linked to toxoplasmosis [29]. The role of water that may contain infective *Toxoplasma* oocysts and thereby contaminate fruit or vegetables during growth is important. Lass et al. [28] reported the contamination of leafy greens with *T. gondii* oocysts. Unwashed vegetables and fruits or washing with contaminated water could increase risk of *T. gondii* infection; however, information on the pathways and risk profile of different produce is limited at this stage.

## 4. Reducing Foodborne *T. gondii* Infections

Data on the frequency, severity and duration of symptoms of human toxoplasmosis are crucial to improve the determination of the burden of the disease which could lead to more adequate prevention strategies. To prevent foodborne *T. gondii* infections, several precautions have been suggested [10,13]:

(1) Good hygiene practices should be always observed i.e., frequent washing of hands or wearing of gloves when in contact with soil or feces from cats and other animals.
(2) Surface water should be boiled before drinking to kill oocysts.
(3) Meat should be cooked to safe temperatures. Beef, lamb, and veal roasts and steaks should be cooked to at least 63 °C, and pork, ground meat, and wild game should be cooked to 71 °C before eating. Whole poultry should be cooked to 82 °C in the thigh to ensure doneness.
(4) Use of unpasteurized goat’s milk should be avoided.
(5) Fruits and vegetables should be peeled or thoroughly washed before eating.
(6) Cutting boards, dishes, counters, utensils, and hands should always be washed with hot soapy water after they have had contact with raw meat, poultry, seafood, or unwashed fruits or vegetables.
(7) Pregnant women should wear gloves when gardening and during any contact with soil or sand because cat waste might be in the soil or sand. After gardening or contact with soil or sand, hands should be thoroughly washed.

It is important to note that infection with *T. gondii* is increasingly being recognized as a problem in non-pregnant, immunocompetent adults, where acute infection may lead to impaired eyesight. Therefore, the focus of research and education must be expanded from infection during pregnancy to human infections in general [7]. Strategies to reduce the prevalence in farm animals include cat control, rodent control, indoors production systems, and decontamination of animal feed and bedding.

## 5. Conclusions

Toxoplasmosis is a serious illness in a relatively small proportion of those exposed to the parasite. There is still much to be learned about the routes of human infection. This is in part due to the complexity of the parasite’s life cycle and in part due to the high proportion of illnesses that result from sporadic infection, which are difficult to investigate. However, the consumption of raw or undercooked meat containing *T. gondii* tissue cysts, the consumption of raw vegetables contaminated with oocysts from cat feces, and water also contaminated with *T. gondii* oocysts from cat feces contribute to human illness caused by this organism. Thorough cooking of meats, avoiding unpasteurized goat’s milk, and observing general good hygiene practices would reduce the risk of foodborne toxoplasmosis.

## Figures and Tables

**Table 1:** Comparative burden of disease estimates for *T. gondii* and other foodborne pathogens from the Netherlands and the USA.

	DALY (Netherlands 2009)	Estimated Cost of Illness in $ million (USA 2009)	Mean QALY Loss (USA 2009)
*Campylobacter* spp.	3250	1747	13,256
*Salmonella* spp. (non-typhoidal)	1270	3309	16,782
Norovirus	1480	2002	5027
*T. gondii* (congenital)	2270	122	1642
*T. gondii* (acquired/adult)	1350	2852	9323
References	[8]	[9]	

DALY, Disability Adjusted Life Years; QALY, Quality Adjusted Life Years.

**Table 2:** Reports on the role of food vehicles in the transmission of toxoplasmosis to humans.

Food Type	Risk Level	Reference
Meat	Pork: The combination of *Toxoplasma* and undercooked pork ranked second among 10 pathogen-food combinations.	[23]
	Sheep: The prevalence of *T. gondii* in sheep is higher than lamb (<1-year-old).	[24]
	Goat: *T. gondii* prevalence in goat meat is not well defined due to the limited number of studies. The contamination level is lower than sheep due to difference in grazing patterns.	[12]
	Cattle: They are considered a poor intermediate host for *T. gondii*.	[25]
	Poultry: Chicken is considered an important host in the epidemiology of *T. gondii* infections.	[26]
	Organic/free-range: The prevalence of *T. gondii* in organic livestock and free-range poultry is significantly higher than animals raised by other methods due to free access to the outdoors and grazing pastures.	[26]
Unpasteurized goat’s milk	Drinking of unpasteurized goat’s milk poses an elevated risk of *T. gondii* infection in human.	[27]
Fresh plant products	Fresh plant products that are unwashed may be contaminated with oocysts from soil, or they may be washed using contaminated water.	[28]
Water	Drinking of contaminated water or use of such water in food preparation.	[29]
Raw seafood	Eating raw oysters, clams, or mussels are emerging risk factors.	[30]
